# Corona Virus Disease 2019 (COVID-19): the image tells the truth

**DOI:** 10.1007/s15010-020-01431-6

**Published:** 2020-05-08

**Authors:** Pei-lun Han, Tong Pang, Kai-yue Diao, Zhi-gang Yang

**Affiliations:** Department of Radiology, West China Hospital, Sichuan University, No. 37 Guoxue Xiang, Chengdu, 610041 China

A 34-year-old male came to our hospital on 21st Jan and complained of fever (peak temperature: 38.5 °C). He lived in Wuhan for the past years and came to visit his relatives in Chengdu for the Spring Festival. He reported no exposure to the “South-China Seafood Market” in Wuhan during the recent months. The fever presented on 20th Jan. He took some Cefoperazone but was not relieved.

**Fig. 1:**
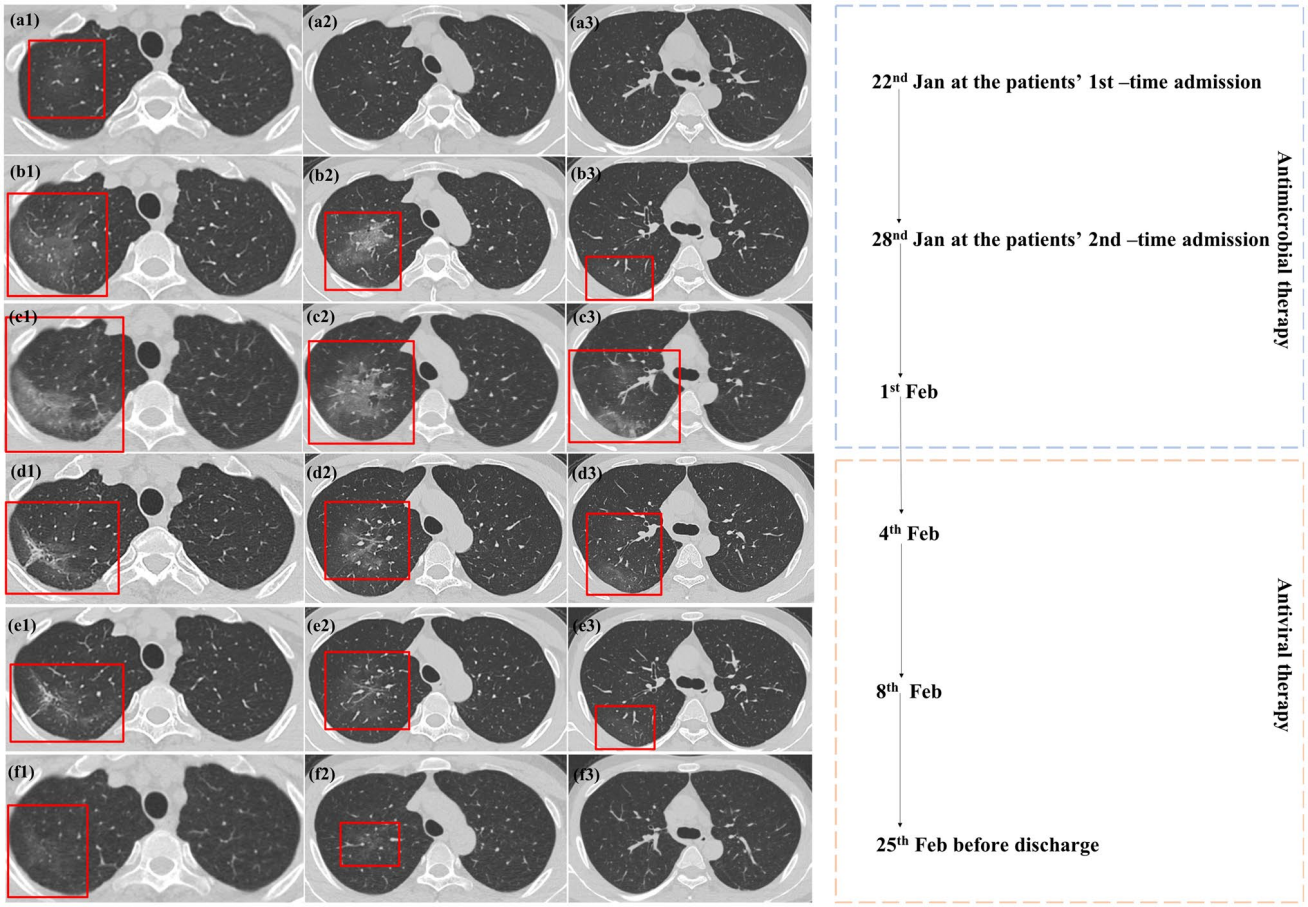
Comparison between the initial and follow-up CT scans. **a1**–**a3** were on 22nd Jan at the patient’s 1st-time admission. **b1**–**b3** were on 28th Jan at the patient’s 2nd-time admission with the same level of **a1**–**a3** separatory. **c1**–**c3** were on 1st Feb after antimicrobial therapy. **d1**–**f3** were CT scans during antiviral therapy. **a1** showed lightly ground-glass opacity (GGO) located at the right apical segment, which was prominently enlarged on **b1** and **b2**, **b3** showed newly increased GGO, as well as evidence of fibrosis (**b2**). **c1**–**c3** showed enlarged GGO and fibrosis after antimicrobial therapy. **d1** shows newly increased fibrosis, which was reduced in **e1** and disappeared in **f1**. **d1**–**f3** showed that the lesions gradually decreased after antiviral therapy

On admission, the patient had normal lymphocytes and white blood cell count but slightly increased monocyte percentage (17.0%) (Table 1). The high-resolution chest computed tomography (HRCT) showed a mild ground-glass opacity (GGO) at right upper lobar. (Fig. 1a1). Fungal and other common respiratory viral infections were excluded though laboratory test. With suspicion of the Corona Virus Disease 2019 (COVID-19) infection, the throat swab was obtained from the patient for real-time reverse transcription polymerase chain reaction (RT-PCR) assay. The RT-PCR test kits were manufactured by Sansure Biotech Inc. (Changsha, China) and its limit of detection (LOD) was established at a Ct of 40. Before the result came out, the patient was isolated and treated with Cefoperazone continuously. On 25th Jan, his first and second SARS-CoV-2 PCR results were negative; thus, the patient was discharged.

**Table 1:** Clinical symptoms and blood parameter results

Items	First-time admission	Second-time admission
Symptoms	Fever	Fever, cough
Temperature (°)	38.5	37.6
Heart rate	112	97
Respiration rate	38.2	18
Blood pressure (mmHg)	135/109	142/88
*Blood test*		
Partial pressure of oxygen (%)	98	96
Red blood cell count (× 10^12^/L)	4.73	4.01
Hemoglobin (g/L)	148	125
Mean corpuscular hemoglobin (pg)	31.3	31.2
Mean corpuscular hemoglobin concentration (g/L)	350	354
Red blood cell volume distribution width-CV (%)	11.9	11.2
Red blood cell volume distribution width-SD (fL)	39.2	36.0
Platelet count (× 10^9^/L)	170	124
White-cell count (× 10^9^/L)	6.01	2.87
Neutrophils count (× 10^9^/L)		1.02
*Absolute value (× 10*^*9*^*/L)*		
Neutrophils	3.37	1.02
Lymphocytes	1.62	1.32
Monocytes	1.02	0.50
Eosinophils		0
Basophils		0
*Differential blood cell count (%)*		
Neutrophils	56.0	35.6
Lymphocytes	27.0	46.0
Monocytes	17.0	17.4
Eosinophils		0
Basophils		0

*CV* coefficient of variation, *SD* standard deviation

However, the patient continuously felt ill and the fever lasted. He came to our hospital again for further treatment. On this admission, the re-arranged blood test result showed reduced total white blood cell count of 2.87 × 10^9^/L, reduced neutrophil count of 1.02 × 10^9^/L, and normal lymphocytes count. The second HRCT showed that the original GGO grew larger with evidence of fibrosis (Fig. 1b1–b3). Empirical antimicrobial treatment with moxifloxacin failed to resolve the infection (Fig. 1c1–c3).

A repetitive throat swab was obtained again and was sent for SARS-CoV-2 PCR assay. Two days later, the result came back and was positive. The patient was isolated and treated with antiviral therapy (lopinavir/ritonavir and interferon) and anti-inflammatory (glucocorticoid) therapy. On 28th Feb, with reduction of both GGO and fibrosis on HRCT (Fig. 1d1–f3), and twice negative SARS-CoV-2 PCR assay results of throat swabs and stool sample, the patient was discharged. At a telephone follow-up 28 days after the discharge, the patient reported no recurrent symptoms or any other discomfort.

Current guidelines [1] recommended SARS-CoV-2 PCR assay as gold standard testing for COVID-19. However, SARS-CoV-2 PCR could not reflect viral load, and the sampling deviation added to the dissatisfactory sensitivity. Our case showed the ability of chest HRCT to recognize infected patient at the very early stage. Although we cannot completely exclude the possibility of bacterial superinfection to cause the GGO [2, 3], considering the white cell/neutrophil depletion at the second blood test, the progression of lesions on CT after antimicrobial therapy did not support it. According to our case, patients with exposure to the epidemic area and suspicious HRCT findings should be isolated rigorously. Besides, since nasopharyngeal swab was reported to be more sensitivie than throat swab [4], samples from multiple sites might be required to avoid sampling bias for PCR assay.
